# Differential Regulation of Myeloid-Derived Suppressor Cells by *Candida* Species

**DOI:** 10.3389/fmicb.2016.01624

**Published:** 2016-10-13

**Authors:** Anurag Singh, Felipe Lelis, Stefanie Braig, Iris Schäfer, Dominik Hartl, Nikolaus Rieber

**Affiliations:** ^1^University Children’s Hospital and Interdisciplinary Center for Infectious Diseases, University of TübingenTübingen, Germany; ^2^Department of Pediatrics, Kinderklinik München Schwabing, StKM GmbH und Klinikum rechts der Isar, Technische Universität MünchenMunich, Germany

**Keywords:** *Candida*, anti-fungal immunity, myeloid-derived suppressor cells, MDSCs, T-cell suppression, Dectin-1, Dectin-2

## Abstract

Myeloid-derived suppressor cells (MDSCs) are innate immune cells characterized by their ability to suppress T-cell responses. Recently, we demonstrated that the human-pathogenic fungi *Candida albicans* and *Aspergillus fumigatus* induced a distinct subset of neutrophilic MDSCs. To dissect *Candida*-mediated MDSC induction in more depth, we studied the relative efficacy of different pathogenic non-*albicans Candida* species to induce and functionally modulate neutrophilic MDSCs, including *C. glabrata, C. parapsilosis, C. dubliniensis*, and *C. krusei*. Our data demonstrate that the extent of MDSC generation is largely dependent on the *Candida* species with MDSCs induced by *C. krusei* and *C. glabrata* showing a higher suppressive activity compared to MDSCs induced by *C. albicans.* In summary, these studies show that fungal MDSC induction is differentially regulated at the species level and differentially affects effector T-cell responses.

## Introduction

*Candida* species cause one of the most prevalent fungal infections worldwide (Pfaller and Diekema, 2007; Brown et al., 2012). Among various *Candida* species, *Candida albicans* has been the model organism for the most research studies focused on immunity against *Candida* infections (Papon et al., 2013). However, the genus *Candida* consists of multiple species that show a considerable variation in terms of their virulence and phenotype and recent studies showed that particularly diseases caused by NAC species are on the rise (Merseguel et al., 2015).

While *C. albicans* is well characterized in terms of recognition through PRRs mainly CLRs like Dectin-1, Dectin-2, mannose receptor (MR) and Mincle (Brown, 2010; Plato et al., 2015), recognition of NAC species is less precisely defined. In contrast to *C. albicans*, phagocytosis of *C. parapsilosis* by neutrophils was not impaired following Dectin-1 blockade *in vitro* (Linden et al., 2010) and, *dectin-1^-/-^* bone marrow macrophages showed no defect in binding to *C. glabrata* (Kuhn and Vyas, 2012). Interestingly, studies indicated that Dectin-2 also played a more important role in *C. glabrata* infection than Dectin-1 (Ifrim et al., 2014). There is also some evidence that T-cell responses are differentially involved in immunity to NAC species. For example, *C. albicans* and *C. parapsilosis* were shown to induce different T-cell responses (Tóth et al., 2013), but underlying mechanisms by which different *Candida* species exert a differential immune response remained elusive.

Myeloid-derived suppressor cells are characterized by their ability to suppress T-cell responses and have mainly been studied in cancer (Bronte, 2009; Gabrilovich and Nagaraj, 2009). However, expansion and involvement of MDSCs has also been reported during various infectious disease conditions, such as polymicrobial sepsis, tuberculosis, and *Staphylococcus aureus* infections (Delano et al., 2007; Du Plessis et al., 2013; Tebartz et al., 2014). Recently, we showed that *C. albicans* induces a distinct subset of neutrophilic myeloid-derived suppressor cells (G-MDSCs) which is mediated by a Dectin-1/CARD9 signaling pathway, leading to dampening of T-cell and NK-cell responses (Rieber et al., 2015).

To further broaden our understanding of how MDSCs play a role in modulating the host immune response to *Candida* infections, we studied the relative efficacy of different pathogenic NAC species to induce neutrophilic MDSCs, including *C. glabrata, C. krusei*, *C. parapsilosis*, and *C. dubliniensis*.

Our data demonstrate that the generation of MDSCs is largely dependent on the *Candida* species and morphotype. Further results also show, that Dectin-1 but not Dectin-2 has an important role during NAC induced MDSC generation.

## Materials and Methods

### Study Subjects

The study was conducted at the University Children’s Hospital Tübingen (Germany). MDSCs were analyzed in primary cell cultures from peripheral blood obtained from healthy subjects. Informed consent was obtained from all subjects included in the study and the local ethics committee approved all study methods. At the time of blood sampling, all healthy subjects were without any signs of infection, inflammation, or respiratory symptoms.

### *Candida* Species and Culture Conditions

*Candida albicans*, *C. krusei*, *C. glabrata*, *C. dubliniensis*, and *C. parapsilosis* strains were stored as frozen stocks in 35% glycerol at -80°C and routinely grown on Sabouraud (Sab) agar (1% mycological peptone, 4% glucose, and 1.5% agar) and YPD agar (1% yeast extract, 2% bacteriological peptone, 2% glucose, and 1.5% agar) plates at 25°C. One colony was inoculated and shaken at 150 rpm at 30°C in YPD broth (1% yeast extract, 2% bacteriological peptone, and 2% glucose) overnight. Cells were harvested by centrifugation and washed twice in sterile Dulbecco’s phosphate-buffered saline (PBS). Cells were counted in a haemocytometer and density was adjusted to the desired concentration in either PBS or RPMI 1640 medium. To generate hyphae, live yeast forms of *C. albicans* were grown for 6 h at 37°C in RPMI 1640 medium (Gibco-BRL). Heat-inactivated *Candida* cells were prepared by heat treatment of the cell suspension at 90°C for 30 min.

### *In vitro* MDSC Generation and Flow Cytometry

Human MDSCs were generated *in vitro* as described previously (Lechner et al., 2010; Rieber et al., 2015). In brief, isolated human PBMCs were cultured in 24 well flat-bottom plates (Corning) or 25 cm^2^ flasks (Greiner Bio-One) at 5 × 10^5^ cells/ml in RPMI 1640 supplemented with 10% heat-inactivated FCS (PAA Laboratories), 2 mM glutamine (Sigma-Aldrich), 100 IU/ml penicillin, and 100 mg/ml streptomycin (Biochrom; referred to as “complete medium”) for 6 days, and GM-CSF (10 ng/ml, Genzyme), heat-inactivated *C. albicans*, *C. glabrata*, *C. krusei*, C. *dubliniensis*, and *C. parapsilosis* were added at a ratio of 1:5 (Fungi:PBMC) as indicated in figures. Dectin-1 antagonist Laminarin obtained from *Laminaria digitata* (100 μg/ml, Sigma) and Dectin-2 antagonist whole mannan particle preparation isolated *from Saccharomyces cerevisiae* (100 μg/ml, Sigma) were added in cell culture where indicated. For ROS inhibition assays, PBMCs were incubated with NADPH oxidase inhibitor DPI (DPI, 0.1 μM; Sigma-Aldrich) for 1 h prior to adding the stimulants.

The number of MDSCs in % of all cells in medium only cultures was set to 1-fold for every single experiment. The MDSC induction due to the specific stimuli is presented as x-fold compared to medium control. Medium and supplements were refreshed on day 4 and supernatants were frozen for ELISA. After 6 days, all cells were collected from PBMC cultures using non-protease cell detachment solution Detachin (Genlantis). G-MDSCs were characterized as CD33^+^CD11b^+^ CD14^-^ cells as described before (Rieber et al., 2013, 2015).

### Cell Isolation and T-Cell Suppression Assays

For functional assays, CD33^+^ MDSCs were isolated from *in vitro* cultures using anti-CD33 magnetic microbeads and autoMACS^®^Pro Separator (Miltenyi Biotec) according to manufacturer’s instructions. Morphology of the MDSCs was analyzed by cytospin staining. For cytospin stainings 5 x 10^4^ CD33^+^ cells were centrifuged in a Cytospin three centrifuge (Shandon) at 800 rpm for 15 min followed by staining with May-Grunwald-Giemsa method (Supplementary Figure S1). T-cell suppression assays were performed as described previously (Rieber et al., 2015). PBMCs were obtained from healthy volunteers and stained with CFSE according to the manufacturer’s protocol (Invitrogen). PBMCs were stimulated with 100 U/ml IL-2 (R&D Systems) and 1 μg/ml OKT3 (Janssen Cilag). Cell number was adjusted to 5 × 10^5^ cells per ml and a total of 60,000 PBMCs per well were seeded in RPMI1640 (Biochrom) medium, in a 96-well microtitre plate and different numbers of MDSCs in RPMI1640 were added to get an MDSC:T-cell ratio 1:2, 1;4, 1:8, 1:16, and 1:32. The cell culture was supplemented with 10% heat-inactivated human serum, 2 mM glutamine, 100 IU/ml penicillin, and 100 mg/ml streptomycin. After 96 h of incubation in a humidified atmosphere at 37°C and 5% CO2, cells were harvested and supernatants were frozen in -20°C. CFSE-fluorescence intensity was analyzed by flow cytometry to determine T-cell proliferation.

### Flow Cytometry

Antibodies against human CD4, CD8, and CD14 were purchased from BD Pharmingen. Antibodies against CD11b and CD33 were purchased from Miltenyi Biotec. Flow cytometry was performed using a FACSCalibur (BD). Results were expressed as percent of positive cells and mean fluorescence intensity (MFI). Calculations were performed with BD CellQuest Pro analysis software and FlowJo.

### Cytokine Analysis in Culture Supernatants

IL-1β (R&D systems) and GM-CSF (Biolegend) ELISA Kits were used to quantify cytokine protein levels in cell culture supernatants. Released IFN-γ protein was quantified by using the Human IFN-γ DuoSet (R&D Systems). All assays were performed according to the manufacturer’s recommendations.

### Statistical Analysis

Statistical analysis was performed in GraphPad Prism version 6.0 using a one-sample *t*-test. In all tests, differences were considered significant at *P* < 0.05 (^∗^*P* < 0.05; ^∗∗^*P* < 0.01; ^∗∗∗^*P* < 0.001; ^∗∗∗∗^*P* < 0.0001).

## Results

### Different *Candida* Species Induce Functional G-MDSCs

**FIGURE 1:**
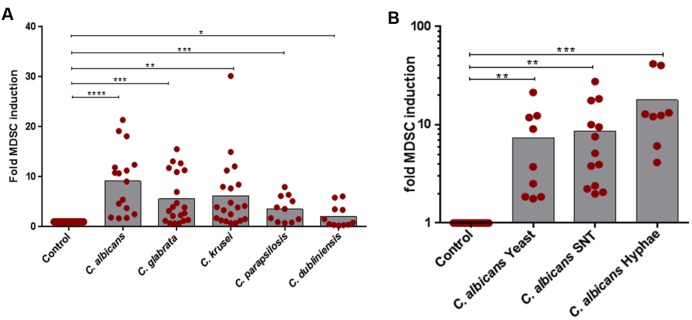
***In vitro* MDSC generation by different *Candida* non-*albicans* species and *C. albicans* morphotypes.** MDSCs were generated by incubating freshly isolated PBMCs (5 × 10^5^/ml) from healthy donors with medium only (negative control) or indicated stimulants. **(A)** PBMCs were cultured with heat killed yeast cells of *C. albicans, C. glabrata, C. krusei, C. parapsilosis*, and *C. dubliniensis* (1 × 10^5^/ml) for 6 days (*n* = 11–20) or **(B)** with heat killed *C. albicans* yeast cells (1 × 10^5^/ml), filter sterilized *C. albicans* yeast supernatant (5% SNT), or *C. albicans* hyphae (1 × 10^5^/ml) for 6 days (*n* = 8–13). Granulocytic MDSCs (CD11b^+^CD33^+^CD14^-^) were quantified by using Flow Cytometry. The number of MDSCs in % of all cells in medium only cultures was set to 1-fold for every single experiment. The MDSC induction due to specific stimuli is presented as x-fold compared to medium control (mean ± SEM) and differences compared to controls were analyzed by a one-sample *t*-test. Significant differences between control and G-MDSCs induction by stimulants are indicated by an asterisk (^∗^*P* < 0.05; ^∗∗^*P* < 0.01; ^∗∗∗^*P* < 0.001; ^∗∗∗∗^*P* < 0.0001).

First, we assessed the ability of NAC species to induce human G-MDSCs and to control their function. G-MDSCs were defined by their surface markers (CD11b^+^CD33^+^CD14^-^) and by their characteristic to suppress T-cell responses. By comparing *Candida* species, we found a differential pattern of MDSC induction among all *Candida* species. While *C. albicans* (9.1-fold) was the strongest inducer of G-MDSCs, *C. krusei*, and *C. glabrata* (5.5- and 6.1-fold, respectively) also induced high amounts of MDSCs, followed by *C. parapsilosis* (3.5-fold) and *C. dubliniensis* (2.1-fold), which was least potent in comparison to others (**Figure 1A**). G-MDSC induction by *C. albicans* was observed for different fungal morphotypes and even occurred using filter sterilized *C. albicans* yeast supernatants (**Figure 1B**). M-MDSCs (CD11b^+^CD33^+^CD14^+^) were not induced during these culture conditions. (Supplementary Figure S2).

### MDSCs Induced by Non-*albicans Candida* Species Are More Suppressive than MDSCs Induced by *C. albicans*

**FIGURE 2:**
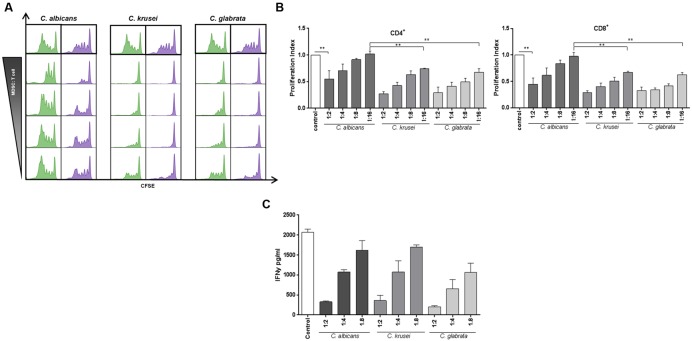
***Candida*-induced MDSCs suppress T cell responses.** MDSCs generated by *Candida* species are able to suppress T-cell proliferation and function in a dose dependent manner. The suppressive effects of CD33^+^-MACS-isolated MDSCs on CD4^+^ (green) and CD8^+^ (lilac) were assessed by T-cell proliferation (CFSE polyclonal proliferation) assay. MDSCs were generated by incubating PBMCs (5 × 10^5^/ml) from healthy donors with heat killed yeast cells of various *Candida* species (1 × 10^5^/ml) or *C. albicans* yeasts for 6 days. **(A)** Representative CFSE stainings, showing the effect of *in vitro C. albicans, C. krusei*, and *C. glabrata* induced MDSCs on CD4^+^ and CD8^+^ T-cell proliferation. Different MDSC to T cell ratios were assessed by using a wide range of MDSC:Target ratio (1:2, 1:4, 1:6, 1:8, and 1:16). **(B)** The bar graphs represent the proliferation index compared to control conditions. Even at a higher MDSC:target ratio of 1:16, MDSCs induced by *C. krusei*, and *C. glabrata* show higher suppressive activity in comparison to *C. albicans*. Data is shown as mean ± SEM (*n* = 4) ^∗∗^*P* < 0.01. **(C)** IFNγ secretion of T cells is decreased by MDSCs. IFNγ secretion in the supernatant was measured on day 4 of MDSC/T cell co-culture experiments by ELISA. The concentration is given in pg/ml (*n* = 3).

The key function attributed to MDSCs is to suppress T-cell responses. (Bronte et al., 2016). Therefore, we performed functional assays to screen for T-cell suppression capability of *Candida*-induced MDSCs. CFSE assays showed that NAS-induced myeloid cells strongly suppressed both CD4^+^ and CD8^+^ T cell proliferation in a dose-dependent manner. Interestingly, MDSCs induced by *C. krusei* and *C. glabrata* exhibited an even higher suppressive activity than MDSCs induced by *C. albicans*, an effect which was significant at MDSC:T cell ratios of 1:8 and 1:16. (**Figures 2A,B**). Apart from T-cell proliferation assays, we also investigated the impact of fungi-derived MDSCs on IL-2 and OKT3-induced T cell cytokine production. These studies demonstrated that MDSCs efficiently suppressed IFN-γ secretion (**Figure 2C**).

### Dectin-1, but not Dectin-2, Is Involved in MDSC Induction by Non-*albicans Candida* Species

**FIGURE 3:**
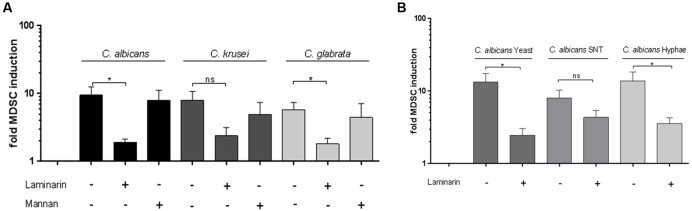
**Dectin-1 is involved in *Candida*-mediated MDSC induction *in vitro*.** MDSCs were generated *in vitro* by incubating isolated PBMCs (5 × 10^5^ cells/ml) with stimulants and inhibitors. **(A)** with heat killed yeast cells of *C. albicans*, *C. krusei*, and *C. glabrata* (all 1 × 10^5^/ml), (*n* = 8–11) or **(B)** with heat killed *C. albicans* yeast cells (1 × 10^5^/ml), filter sterilized *C. albicans* yeast cell supernatant (5% SNT) or *C. albicans* hyphae for 6 days (*n* = 8–13). Where indicated, prior to stimulation, PBMCs were pretreated for 60 min with Dectin-1 inhibitor Laminarin (100 μg/ml) or Mannan (100 μg/ml) from *Saccharomyces cerevisea* to mimic Dectin-2 binding without receptor activating capacity. (^∗^*P* < 0.05, Bars represent SEM).

In our previous work we showed that Dectin-1 plays a key role in *C. albicans*-induced MDSC generation. Several studies also reveal the role of Dectin-1 and also Dectin-2 (Saijo and Iwakura, 2011) in immune mechanisms against NAC species. We therefore focussed on Dectin-1 and Dectin-2 as β-glucan and mannan receptors, essentially involved in recognition of fungi. As shown for *C. albicans*, blocking of Dectin-1 prior to co-culture with fungal cells diminished the MDSC-inducing effect significantly in *C. glabrata*. For *C. krusei*-induced MDSCs we observed a similar, however, not significant effect. On the other hand, blocking of Dectin-2 had no effect (**Figure 3A**) suggesting that Dectin-2 is dispensable for *Candida*-mediated MDSC generation. Since fundamental differences have been reported between host recognition of *C. albicans* morphotypes (Lowman et al., 2014), we next examined the impact of Dectin-1 blockage on MDSC generation. In case of *C. albicans* yeast cells and hyphae, Dectin-1 blockage significantly inhibited the MDSCs. Dectin-1 blockage also led to a similar trend for filter sterilized *C. albicans* yeast cell supernatant, however, it was not significant (**Figure 3B**).

### *Candida*-Mediated MDSC Generation Is Associated with GM-CSF, IL-1β, and ROS Production

**FIGURE 4:**
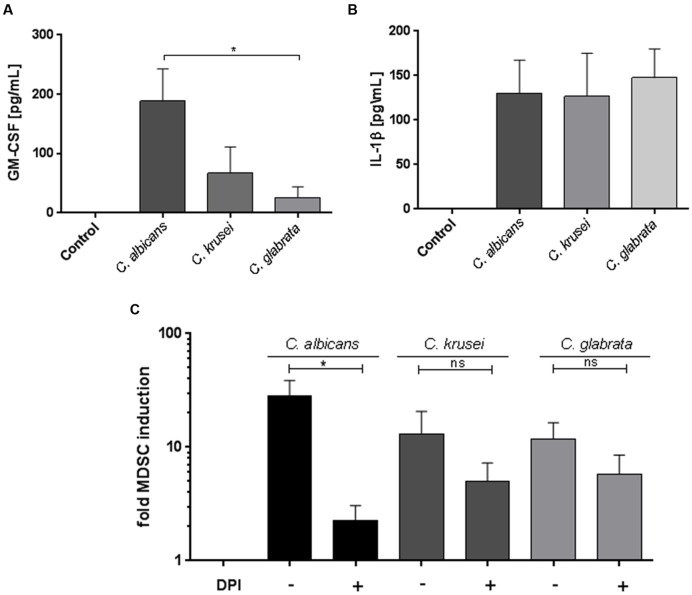
***Candida*-mediated MDSC generation involves GM-CSF, IL-1β, and ROS.** GM-CSF, IL-1β, and ROS are involved in *Candida*-mediated MDSC generation. Freshly isolated PBMCs (5 × 10^5^ cells/ml) were cultured in medium only, or with heat killed yeast cells of *C. albicans* (1 × 10^5^/ml), *C. krusei* (1 × 10^5^ cells/ml), and *C. glabrata* (1 × 10^5^ cells/ml) for 4 days. For quantification of cytokines, co-culture supernatants were collected on day 4. **(A)** GM-CSF (*n* = 8) and **(B)** IL-1β (*n* = 6) levels were quantified by ELISA. **(C)** MDSCs were generated *in vitro* by incubating isolated PBMCs (5 × 10^5^ cells/ml) with with heat killed yeast cells of *C. albicans* (1 × 10^5^/ml), *C. krusei* (1 × 10^5^ cells/ml), and *C. glabrata* (1 × 10^5^ cells/ml) for 6 days. Prior to stimulation, PBMCs were pretreated for 60 min where indicated with the NADPH oxidase inhibitor DPI (0.1 μM; *n* = 6) (^∗^*P* < 0.05, Bars represent SEM).

The cytokine GM-CSF has been involved in MDSC generation (Gabrilovich and Nagaraj, 2009; Dolcetti et al., 2010) and previous studies showed that GM-CSF is secreted upon stimulation with fungal pathogens. (Li and Dongari-Bagtzoglou, 2009; Svobodová et al., 2012). Therefore we hypothesized that GM-CSF might play a role in *Candida*-mediated MDSC generation and analyzed the amount of GM-CSF in conditioned medium obtained from PBMC-*Candida* co-culture. Our results demonstrate that *C. albicans* stimulation leads to a high amount of GM-CSF release in comparison to *C. glabrata* and *C. krusei* (**Figure 4A**). In addition to GM-CSF, the inflammasome product IL-1β has been previously involved in MDSC induction (Elkabets et al., 2010; Lechner et al., 2011; Ballbach et al., 2016). Hence, we quantified IL-1β protein in our assays and found that *C. albicans, C. glabrata*, and *C. krusei*, all three major pathogenic *Candida* species lead to high amounts of IL-1β secretion upon PBMC stimulation (**Figure 4B**). These results indicate that the two MDSC-related cytokines GM-CSF and IL-1β seem to be associated with fungal MDSC induction. ROS have been consistently involved in MDSC generation and function (Gabrilovich and Nagaraj, 2009). To check the role of ROS, MDSCs were generated *in vitro* by incubating isolated PBMCs (5 × 10^5^ cells/ml) with different *Candida* stimulants (1 × 10^5^ cells/ml) and pretreatment for 1 h with the NADPH oxidase inhibitor DPI (0.1 μM) where indicated. These experiments showed that ROS contributed substantially to fungi-mediated MDSC induction *in vitro* (**Figure 4C**).

## Discussion

Previous studies from our group demonstrated that pathogenic fungi *A. fumigatus* and *C. albicans* induce MDSCs, which suppress T cell responses (Rieber et al., 2015). In this study, we compared the capacity of *C. albicans*, *C. glabrata*, *C. krusei*, and *C. parapsilosis* to induce G-MDSCs and the relative strength of *Candida*-induced G-MDSCs to suppress T-cell proliferation and cytokine production.

*Candida* species are found as commensal organisms at mucosal surfaces in the human body. Since *C. albicans* is the most prominent fungus isolated from clinical samples, research related to anti-fungal immune response is largely centered on it. However, recent clinical studies have reported a rise in the NAC species isolated from clinical samples of fungal infections. NAC species associated with disease mainly include *C. glabrata*, *C. krusei*, *C. dubliniensis*, and *C. parapsilosis* (Butler et al., 2009). Here we extend our previous findings by showing that the strength of *Candida*-mediated MDSC induction substantially depends on the *Candida* species. While *C. albicans* was the strongest inducer of MDSCs, *C. dubliniensis* showed the lowest capacity. Importantly, our studies further show that not only the extent, but also the functionality of MDSCs is regulated by distinct *Candida* species. Collectively, these studies add to our understanding of how different *Candida* species differentially modulate host immunity.

*Candida* species consist of a diverse range of virulence factors and morphotypes. Although limited in number, studies using *in vitro* methods and *in vivo* infection strategies demonstrate that host innate immune responses to *Candida* challenge including activation and function of neutrophils (Dementhon et al., 2012; Svobodová et al., 2012; Duggan et al., 2015), dendritic cells (Bourgeois et al., 2011), and macrophages (Seider et al., 2011) differ depending on the *Candida* species. In addition to different species, we also used *C. albicans* yeast and hyphal forms and filter sterilized supernatant from yeast cultures to study the impact of different fungal morphotypes and soluble products during fungi-mediated MDSC generation. *C. albicans* yeast to hyphae morphogenesis has been attributed as a crucial virulence factor during fungal pathogenesis. Various studies demonstrate that immune cell recognition and subsequent immune response toward different morphotypes of *C. albicans* differs (Lewis et al., 2012; Lowman et al., 2014) due to differential exposure of cell wall components, e.g., β-glucans (Wheeler et al., 2008; Gow et al., 2011). However, in our studies, we did not find a difference in MDSC induction after stimulation with *C. albicans* yeast and hyphae (Rieber et al., 2015) or supernatants. Further studies involving various morphotypes of different NAC species and secreted fungal virulence factors will help to dissect the mechanism underlying *Candida*-mediated MDSC generation and function. T cells are pivotal immune cells during *C. albicans* infection and patients with decreased CD4+ T cells were found to be highly susceptible to mucocutaneous and invasive Candidiasis (Fidel, 2011; Lionakis and Netea, 2013). Interestingly, *C. glabrata* and *C. krusei*-generated MDSCs were more suppressive on T cell proliferation than *C. albicans*-generated MDSCs and this phenomenon was recapitulated in the suppression of IFNγ release. There is some evidence suggesting differential T-cell responses depending on the *Candida* species. *C. albicans* and *C. parapsilosis* were found to induce different T-cell responses and cytokines. Human PBMCs stimulated with heat killed *C. parapsilosis* yeast cells showed higher production of IL-10 but lower amounts of IL-1β, IFNγ, IL-17, and IL-22, when compared to cells stimulated with *C. albicans* (Tóth et al., 2013). Another study reported distinct T-cell generation in response to *C. albicans* and NAC species and T cells generated after stimulation with *C. albicans* displayed cross-reactivity only with *C. tropicalis* but not *C. glabrata* (Tramsen et al., 2007). Our findings now also hint toward a species-dependent innate immune response against different *Candida* species. The induction of MDSCs might contribute to a fine-tuned balance between pro-inflammatory effector and counter-regulatory immune mechanisms, which has been demonstrated to be crucial for an effective anti-fungal immune response (Zelante et al., 2011, 2012; Rieber et al., 2015).

*Candida albicans* is recognized by different classes of PRRs among which, the CLRs including Dectin-1and Dectin-2 are the most important ones described so far. In our previous work, we showed that dectin-1 mediated signaling was prominent in fungi-induced MDSC generation. While Dectin-1 has been shown to be the key PRR for *C. albicans* (Taylor et al., 2007; Marakalala et al., 2013), Dectin-2 has emerged as a leading PRR to recognize both *C. albicans* and *C. glabrata* (Saijo et al., 2010; Ifrim et al., 2014). Therefore we focussed on these two PRRs to clarify their role in *Candida*-mediated MDSC generation. In consistence with our previous findings for *C. albicans* (Rieber et al., 2015), we found that blockage of Dectin-1 but not Dectin-2 led to diminished MDSC generation by *C. albicans*, *C. glabrata*, and *C. krusei*. Our results demonstrate that *Candida*-mediated MDSC induction is dependent on the type of *Candida* species, which is in line with the notion that anti-fungal immune responses are species- and strain-specific and vary in terms of recognition by the host immune system (Netea et al., 2010; Marakalala et al., 2013). Future studies will be essential to expand the understanding how differential adaptation of *Candida* strains plays a role in MDSC generation. Different morphotypes of *C. albicans* induce an altered immune response. It has been reported that *C. albicans* yeast cells and hyphae are differentially recognized by Dectin-1 and Dectin-2 during host-pathogen-interaction (Saijo et al., 2010; Saijo and Iwakura, 2011). We observed a similar MDSC induction independent of the *C. albicans* morphotype. Dectin-1 blockage significantly inhibited the MDSC generation by *C. albicans* yeast cells and hyphae, and led to a similar trend for *C. albicans* supernatant. This hints toward the presence of a soluble Dectin-1 ligand in *C. albicans* supernatant that contributes to MDSC generation. Interestingly, while yeast mannan particles have been described to impact not only Dectin-2, but also other PRRs like MR, DC-SIGN, and Mincle (Netea et al., 2015), we did not observe any effect of mannan treatment on *Candida*-mediated MDSC generation in our studies.

To elucidate the mechanism of *Candida*-mediated MDSC induction, we further focused on two key cytokines, GM-CSF and IL-1β, both reported to play an important role in MDSC generation and homeostasis (Elkabets et al., 2010; Lechner et al., 2011; Gabrilovich et al., 2012; Bayne et al., 2016), as well as during fungal pathogenesis (Svobodová et al., 2012; Netea et al., 2015). Stimulation of PBMCs with *C. albicans* and NAC species led to release of GM-CSF and IL-1β. *C. albicans*-mediated release of GM-CSF was significantly higher than that of *C. glabrata*, possibly explaining the stronger induction of MDSCs upon *C. albicans* stimulation. All three species *C. albicans*, *C. glabrata*, and *C. krusei* released similar amounts of IL-1β upon PBMC stimulation. Since Dectin-1 was found to be the key receptor for *Candida*-mediated MDSC generation, and previous studies demonstrated that ROS act downstream of Dectin-1 (Branzk et al., 2014), and ROS have been shown to be involved in MDSC homeostasis (Corzo et al., 2010; Gabrilovich et al., 2012), we further examined the role of ROS for *Candida*-mediated MDSC induction. These studies demonstrated that ROS contributed substantially to NAC-mediated MDSC induction *in vitro*.

## Conclusion

Our results demonstrate that *Candida*-mediated MDSC induction is differentially regulated at the species level and differentially affects effector T-cell responses. In our previous study using a systemic infection mouse model for *C. albicans*, we showed that adaptive transfer with MDSCs leads to a protective effect against invasive Candidiasis. While the classical MDSC inducing factor GM-CSF has already been proposed as one of the leading candidates for anti-fungal adjunctive therapy (Vazquez et al., 1998; van de Veerdonk et al., 2012), *in vivo* generation of MDSCs or *ex vivo* expansion and adoptive transfer might become an interesting approach for future therapeutic strategies against infections caused by *Candida* species.

## Author Contributions

AS designed the study, performed the experiments, analyzed the data, and wrote the manuscript. FL, SB, and IS performed the experiments. DH and NR co-designed the study, supervised experiments, discussed data, and co-wrote the manuscript.

## Conflict of Interest Statement

The authors declare that the research was conducted in the absence of any commercial or financial relationships that could be construed as a potential conflict of interest.

## Abbreviations

CFSE: Carboxyfluoresceinsuccinimidyl ester
DPI: Diphenyleneiodonium chloride
ELISA: Enzyme-linked immunosorbent assay
FACS: Fluorescence-activated cell sorting
GM-CSF: Granulocyte-macrophage colony-stimulating factor
G-MDSCs: Granulocytic myeloid-derived suppressor cells
IL-: Interleukin
MACS: Magnetic-activated cell sorting
MDSCs: Myeloid-derived suppressor cells
M-MDSCs: Monocytic myeloid-derived suppressor cells
NAC: Non-*albicans Candida*
PBMCs: Peripheral blood mononuclear cells
PRR: Pattern recognition receptor
ROS: Reactive oxygen species
