# Supplementary material for: Differential Regulation of Myeloid-Derived Suppressor Cells by Candida Species
Source: Front Microbiol. 2016 Oct 13;7:1624. doi: 10.3389/fmicb.2016.01624 (PMC5061774; doi:10.3389/fmicb.2016.01624)
Supplement: Supplementary file 1 [file Presentation_1.PDF]

Supplementary Figure S1

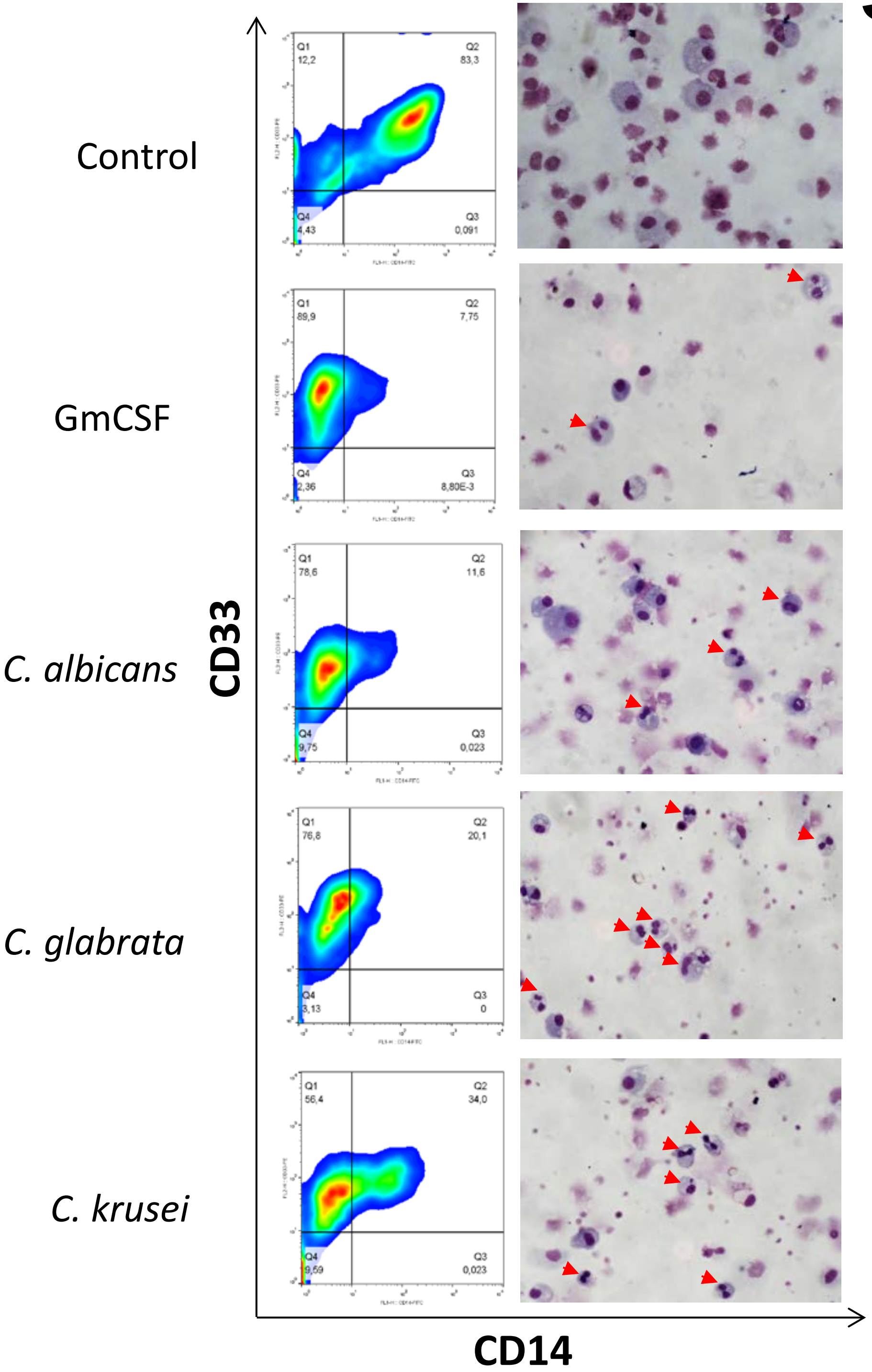

FACS gating and photomicrograph showing granulocytic morphology of *in vitro Candida*-induced MDSCs:
MDSCs were generated *in vitro* by incubating isolated PBMCs ($5x10^5$ cells/ml) with GM-CSF, heat killed yeast cells of *C. albicans*, *C. krusei* and *C. glabrata* (all $1x10^5$/ml) for 6 days. Phenotyping was done by selecting $CD33^+CD14^-$ cells.
For microscopy, $CD33^+$ MDSCs were MACS-isolated after 6 days culture and cytospins were stained with May–Gruenwald–Giemsa. Pictures were obtained by using a reverted Zeiss Axiovision Microscope mounted with a Canon 550D camera. Cells with a granulocytic-MDSC morphology are marked with red arrow.
