# Supplementary material for: Differential Regulation of Myeloid-Derived Suppressor Cells by Candida Species
Source: Front Microbiol. 2016 Oct 13;7:1624. doi: 10.3389/fmicb.2016.01624 (PMC5061774; doi:10.3389/fmicb.2016.01624)
Supplement: Supplementary file 2 [file Presentation_2.PDF]

Supplementary Figure S2

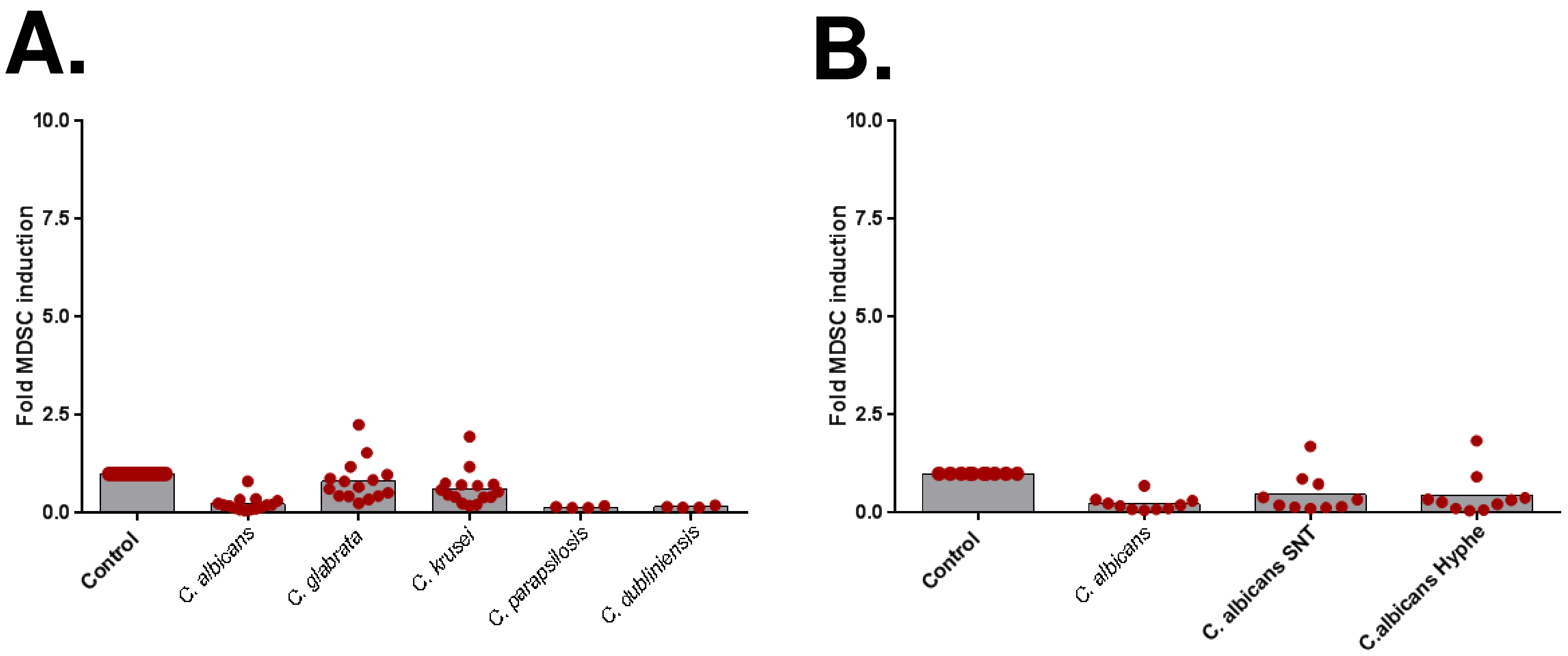

M-MDSCs are not induced after fungal stimulation of human PBMCs:
MDSCs were generated by incubating freshly isolated PBMCs (5 x 10^5 /ml) from healthy donors with medium only (negative control) or indicated stimulants. (A) PBMCs were cultured with heat killed yeast cells of *C. albicans, C. glabrata, C. krusei, C. parapsilosis and C. dubliniensis* (1 x 10^5 /ml) for 6 days (n=4-15) or (B) with heat killed *C. albicans* yeast cells (1 x 10^5 /ml), filter sterilized *C. albicans* yeast supernatant (5% SNT) or *C. albicans* hyphae (1 x 10^5 /ml) for 6 days (n=10). M-MDSCs ($CD11b^+CD33^+CD14^+$) were quantified by using Flow Cytometry. The number of MDSCs in % of all cells in medium only cultures was set to 1-fold for every single experiment. The MDSC induction due to specific stimuli is presented as x-fold compared to medium control (mean ± SEM). No M-MDSCs were found in the cell population.
